# Opinions and Practice of US-Based Obstetrician-Gynecologists regarding Vitamin D Screening and Supplementation of Pregnant Women

**DOI:** 10.1155/2016/1454707

**Published:** 2016-08-25

**Authors:** Sara A. Mohamed, Ayman Al-Hendy, Jay Schulkin, Michael L. Power

**Affiliations:** ^1^Medical College of Georgia, Georgia Regents University, Augusta, GA 30912, USA; ^2^The American College of Obstetricians and Gynecologists, Washington, DC 20024, USA

## Abstract

Vitamin D deficiency/insufficiency is prevalent among pregnant women. Recommendations for adequate levels of circulating 25-hydroxyvitamin D and appropriate vitamin D supplementation during pregnancy differ between the Institute of Medicine and the Endocrine Society. Obstetrician-gynecologists must make clinical decisions in this environment of uncertain guidance. An online questionnaire regarding physician practice patterns for screening and supplementing pregnant women was administered to 225 randomly selected practicing obstetrician-gynecologists of whom 101 (45%) completed the questionnaire. A majority indicated that vitamin D insufficiency was a problem in their patient population (68.4%) and that most of their pregnant patients would benefit from vitamin D supplementation (66.3%). Half (52.5%) would recommend vitamin D supplementation during pregnancy to some patients, but only 16.8% to all. Only one in four (25.8%) routinely screen their pregnant patients for vitamin D status. Physicians who indicated that vitamin D status was a problem in their patient population were more likely to screen routinely (32.8% versus 9.7%, *P* = 0.002) and believe their patients would benefit from supplementation (91.2% versus 16.1%, *P* = 0.001). Opinion regarding supplementation levels and indicators of adequacy were split between the two competing recommendations, suggesting that clinical practice will likely remain variable across physicians, with uncertain public health consequences.

## 1. Introduction

It is only relatively recently in human history that vitamin D became a required nutrient. Vitamin D is produced in skin when exposed to unfiltered sunlight. Our species evolved under circumstances of extensive sun exposure, so in the evolutionary sense vitamin D was not a true vitamin but rather an endogenously photosynthetically produced precursor to the active steroid hormone (1,25-dihydroxyvitamin D). The geographic expansion of humanity, the modern environment, and medical concerns over the sequela of extensive sun exposure (e.g., melanoma) have created conditions where for much of modern humanity endogenous vitamin D production is likely far below that of our ancestors. The importance of dietary vitamin D, both naturally occurring in foods and via supplements added to foods (e.g., fortified milk) or consumed directly, has increased.

Vitamin D has biological actions in support of calcium metabolism and immune function, among many others, important for maternal, fetal, and neonatal health [1, 2]. Poor vitamin D status is a risk factor for poor skeletal development and fractures in newborns [3]. More recently, maternal hypovitaminosis D status has been associated with several pregnancy complications, such as preeclampsia, being small for gestational age, low birth weight, and preterm birth in a number of populations around the world [4–10]. Fetal and neonatal vitamin D status is largely dependent upon maternal status [11], and maternal vitamin D deficiency/insufficiency is distressingly prevalent in the United States. As many as 30% of pregnant women in the US may have inadequate vitamin D status [12]. In some populations, especially during the winter months in higher latitudes, the proportion of pregnant women with inadequate vitamin D status may exceed 50% [13], depending on whose definition of inadequate is used.

There is some uncertainty over the extent of the US population that has inadequate vitamin D status due to disagreements between experts as to what constitutes inadequate status. The main storage form of vitamin D in blood is 25-hydroxyvitamin D (25-OH-D) which makes this vitamin D metabolite the best indicator of nutritional status [2]. Based on data regarding bone health, the Institute of Medicine panel [14] concluded that a circulating level above 20 ng/mL of 25-OH-D is sufficient and found no evidence for recommending higher values for any subpopulation, including pregnant women [15, 16]. Other researchers have questioned this finding, citing both a concern that circulating levels of 25-OH-D at 20 ng/mL have not been shown to be sufficient for bone health for all populations and that other nonskeletal functions of vitamin D potentially important for health, which the IOM report discounts based on inadequate evidence, may yet be shown to be important and require higher circulating levels of 25-OH-D [17, 18]. The Endocrine Society defined deficiency as circulating 25-OH-D of less than 20 ng/mL but also defined 20–29 ng/mL as insufficiency, with a recommended level of 25-OH-D above 30 ng/mL [19]. The two groups also recommend different levels of vitamin D supplementation, in large part due to the different stable levels of circulating 25-OH-D they are attempting to obtain.

Low to moderate quality evidence exists for a protective effect of vitamin D supplementation during pregnancy for a number of pregnancy outcomes, including preeclampsia, being small for gestational age, and preterm labor [20]. Low circulating 25-OH-D levels (below 32 ng/mL) also have been associated with these pregnancy complications, as well as with an increased risk of maternal infectious disease including vaginosis and periodontal disease, both risk factors for preterm birth [21, 22]. Observational studies have consistently found an association with low maternal vitamin D status and increased risk of neonatal infectious disease and later inflammatory and atopic disease in offspring [21]. In vitro studies have shown that 1,25-dihydroxyvitamin D downregulates gene expression of inflammatory markers and contractile associated proteins in human myometrial cells [23, 24], providing a biologically plausible mechanism for vitamin D action in reducing inflammatory and contractile responses due to infection. However, causality for any of these conditions is yet to be established.

Screening for vitamin D status is relatively easy and accurate [19], though there is a nontrivial cost. Vitamin D supplementation during pregnancy is generally considered safe, though rigorous studies on high doses of vitamin D supplementation are lacking [25]. Results from a recent study indicate that vitamin D supplementation during pregnancy with up to 4,000 IU/day is safe and effective [26]. However, consensus on what constitutes adequate vitamin D status during pregnancy and the most efficacious means to prevent and treat vitamin D deficiency/insufficiency in pregnant women has yet to be reached, leading to differing assessments of this public health issue [14, 19]. Vitamin D status and supplementation of pregnant women is a good example of where obstetrician-gynecologists must make practice decisions in an environment of some uncertainty regarding the risks and rewards. The purpose of this study was to assess the practice, knowledge, and opinions of practicing obstetrician-gynecologists regarding screening for vitamin D status and vitamin D supplementation of their pregnant patients.

## 2. Methods

An online questionnaire survey was administered to a randomly selected group of 225 of the 1,167 Fellows and Junior Fellows in Practice of the American College of Obstetricians and Gynecologists (ACOG) who belong to the Collaborative Ambulatory Care Network (CARN). These Fellows agree to participate in multiple ACOG Research Department studies each year. CARN Fellows have been selected to be demographically representative of practicing Fellows of the College as a whole. The ACOG Research Department actively maintains the CARN member list by targeted recruitment and attrition to minimize demographic differences with ACOG Fellows as a whole. The survey contained questions on physician and patient population demographics, physician opinion regarding health concerns associated with vitamin D deficiency/insufficiency, and practice patterns regarding screening and supplementing pregnant women.

The survey was administered through an online survey site called Real Magnet. Cover letters with a description of the survey were mailed to the potential participants in early April 2015. Three days after cover letters were sent, physicians were emailed an online link to the survey. Four email reminders were sent to nonresponders at approximately two-week intervals. Data collection ended on July 30, 2015.

Statistical analysis was performed using IBM SPSS Statistics 20.0, IBM Corp.^©^, Armonk, NY. We calculated distribution of frequencies for each question in the survey study. Pairwise comparisons of categorical variables were analyzed using the chi-square test of association. We used two-sided statistical inferences and a significance level of a *P* value of 0.05 or less.

## 3. Results

Of the potential 225 study subjects, 101 completed the questionnaire. Among nonrespondents, 69 declined to participate with no comment, 43 made no response at all, 7 declined and stated they were not eligible (did not treat pregnant patients), 1 had a technical difficulty with the survey link and was unable to participate, and 4 returned incomplete questionnaires and were excluded. After excluding the ineligible recipients and the recipient with the technical difficulty, the response rate was 46.5% (101/217). Respondents came from all across the United Sates, with practices in 37 states and the District of Columbia. There were no differences in responses between physicians in states mostly south of the 35th parallel versus those mostly north of that latitude. The responding physicians estimated the racial/ethnic makeup of their patient population to be, on average, 49.9% non-Hispanic white, 18.2% Hispanic, 18.0% non-Hispanic African American, 5.9% Asian/Pacific Islander, 2.9% Native American, and 5.2% multiracial.

The demographic data for the responding physicians can be found in Table 1. The sample was comprised primarily of non-Hispanic whites (83.2%). Most respondents rated primary care as very important (38.6%) or important (41.6%) to their practice. Less than half (45.5%) had read ACOG Committee Opinion 495 (CO 495) on vitamin D during pregnancy, and opinion was split evenly among these physicians on whether this committee opinion had changed their practice regarding vitamin D screening and supplementation during pregnancy. A majority responded that vitamin D insufficiency was a problem in their patient population (68.3%) and that most of their patients would benefit from taking a vitamin D supplement (66.3%). Geographic location of the practice and racial/ethnic makeup of the patient population had no effect on these opinions.

Fortified dairy products were the vitamin D source recommended by most respondents, followed by green leafy vegetables, fatty fish, fortified cereals, and vitamin D supplements and multivitamins. Sun exposure was recommended by about two of three respondents; almost none recommended an artificial UV light source (Table 2).

A majority of respondents considered vitamin D supplementation during pregnancy (82.1%) to be safe. Respondents were concerned about vitamin D deficiency and osteoporosis (91.1%) and bone fractures (88.1%), but less concerned about other possible sequelae of vitamin D deficiency such as a weakened immune system (32.7%), colon cancer (27.7%), cardiovascular disease (23.8%), impaired glucose metabolism (22.8%), vulnerability to preeclampsia (22.8%), increased risk of small-for-gestational-age infant (21.8%), vulnerability to preterm labor (19.8%), breast cancer (14.9%), hypertension (12.9%), and infertility (10.9%).

More than half of the respondents indicated that they never (32.7%) or rarely (23.8%) screen their pregnant patients for vitamin D; about one in four always (12.9%) or often (12.9%) screen. More respondents disagreed (41.6%) than agreed (22.8%) that all pregnant women should be screened for vitamin D deficiency. Physicians that reported having read CO 495 were more likely to screen their pregnant patients (36.9% always or often versus 17.0% that had not read CO 495; *P* < 0.001) as were physicians that reported that vitamin D insufficiency was a problem in their patient population (32.8% always or often versus 9.7%; *P* = 0.004). Physicians with 27 or more years in practice were more likely to screen their pregnant patients (31.3% always or often versus 21.8%; *P* = 0.023). Physician geographic location and patient population were not significant factors.

A majority of the responding physicians agreed that alcohol abuse (79.2%) and African American race (51.5%) were risk factors for vitamin D insufficiency. Almost half (49.5%) agreed that obese women were at higher risk. Patient factors that would increase the likelihood that a physician would screen for vitamin D status included evidence of malabsorption syndrome (82.2%), gastric bypass surgery (79.2%), family history of osteoporosis (75.8%), and evidence of alcohol abuse (56.4%). Obesity (34.6%) and African American race (28.8%) had less of an effect on physician inclination to screen.

Almost all respondents recommend that pregnant patients be prescribed or counseled to take prenatal vitamins (92.1% always and 3.0% often). There was a lack of consensus of opinion among respondents regarding whether pregnant women taking prenatal vitamins are at low risk of vitamin D deficiency, with equal proportions agreeing and disagreeing and the modal response being neutral. Most respondents were either neutral (23.8%) or disagreed (41.6%) with the following statement: Most of my pregnant patients will get enough vitamin D through sun exposure and diet. About half of respondents recommend additional vitamin D supplementation to their pregnant patients (always = 16.8%, often = 11.9%, and sometimes = 23.8%). Physicians who considered vitamin D insufficiency a problem among their patients were more likely to think that most of their pregnant patients would benefit from taking vitamin D supplements (91.2% versus 16.1%; *P* < 0.001). The most common answer for vitamin D supplementation dose given to a pregnant woman with no sign of vitamin D insufficiency was 1,000 IU/day (41.6%), followed by 400 IU/day (22.8%) and 600 IU/day (20.8%). A few respondents would recommend 1,200 IU/day (5.0%), 2,000 IU/day (3.0%), or 4,000 IU/day (2.0%) for all pregnant women. Physicians with 27 or more years in practice were more likely to recommend 1,000 or 1,200 IU/day (67.8% versus 38.1%; *P* = 0.033). Regarding the level of circulating 25-hydroxyvitamin D below which there is concern for vitamin D deficiency, respondents were roughly split between below 20 ng/mL (50 nmol/L) and 32 ng/mL (80 nmol/L) as the level at which they would recommend a higher supplementation dose of vitamin D (46.5% versus 40.6%). A few respondents (5.9%) indicated enhanced concern at 50 ng/mL (125 nmol/L). There was no effect of physician or patient population demographics on the distribution of these answers.

Physicians who indicated that vitamin D insufficiency is a problem in their patient population were more likely to agree or strongly agree that vitamin D supplementation during pregnancy is safe and that all pregnant women should be screened. However, even among this group, only about one of three agreed that all women should be screened (Table 3). These physicians were also more likely to disagree with the following statements: vitamin D supplementation during pregnancy is generally not necessary, they are not concerned about vitamin D deficiency in their pregnant patients, pregnant women taking prenatal vitamins are at low risk for vitamin D deficiency, and most of my pregnant patients will get enough vitamin D through sun exposure and diet (Table 3).

## 4. Discussion

The results of this study suggest that practicing obstetrician-gynecologists generally are somewhat concerned about the vitamin D status of their pregnant patients and consider vitamin D supplementation during pregnancy to be safe. The greater the concern is over their patients' vitamin D status, the more likely the physician is to recommend supplementation. The respondents seemed generally knowledgeable regarding conditions that increase the risk of vitamin D insufficiency, such as malabsorption syndrome, gastric bypass surgery, alcohol abuse, African American race, and obesity. The responding physicians were generally not supportive of screening all pregnant women for vitamin D status; even among physicians that expressed a concern regarding their patient population, less than half would screen most pregnant patients.

There was a lack of consensus regarding the appropriate level of vitamin D supplementation during pregnancy and the value of circulating 25-hydroxyvitamin D, below which there is concern regarding vitamin D insufficiency. The lack of consensus among clinicians mirrors a lack of consensus among researchers and recommendations from authoritative bodies. There is continuing controversy over the appropriate levels of vitamin D supplementation and of the levels of circulating 25-hydroxyvitamin D that represent good health. The result has been conflicting recommendations from the IOM [14, 15] and the Endocrine Society [19], especially concerning vitamin D supplementation and appropriate levels of circulating 25-OH-D during pregnancy (Table 4).

In support of the Endocrine Society recommendations, some researchers point to the likely higher circulating levels of 25-OH-D in our ancestors that relied primarily on photosynthetic production of vitamin D, arguing that our vitamin D metabolism is adapted to high endogenous production [17, 18]. For example, circulating levels of 25-OH-D in traditionally living people in Tanzania (mean 44 ng/mL; range of 23–69 ng/mL) were double the IOM suggested 20 ng/mL value for sufficiency [27].

Vitamin D metabolism differs during pregnancy, with a substantial increase in the production of the active form (1,25-dihydroxyvitamin D), likely due in part to placental production, resulting in elevated levels relative to the nonpregnant state [2, 28]. There is also a direct association between circulating 25-OH-D and 1,25-OH2-D, which is not the case outside of pregnancy, suggesting both higher substrate (25-OH-D) turnover and a greater effect of 25-OH-D levels on vitamin D actions on physiology and metabolism [2]. Low circulating 1,25-OH2-D is associated with preterm birth [8]. The association between 25-OH-D and 1,25-OH2-D appears to plateau above 40 ng/mL of 25-OH-D [26], leading some researchers to conclude that the Endocrine Society recommended levels for circulating 25-OH-D are more metabolically appropriate during pregnancy (e.g., [18]). Even researchers skeptical of the causality of vitamin D effects on pregnancy outcomes point to a knowledge deficit that needs addressing, especially in areas such as maternal and neonatal infections (e.g., [21]).

The levels recommended by IOM have been called too conservative and driven by a concern regarding health risks of oversupplementation and high circulating levels of 25-OH-D for which there is scant evidence and that appear implausible from an evolutionary perspective [17]. On a cautionary note, the evidence for positive benefits of circulating levels of 25-OH-D and vitamin D supplementation above those from IOM can hardly be called definitive. A recent study on a mother-offspring cohort in Singapore found no evidence for an effect of vitamin D status on birth outcome, though the authors caution that the population had a low prevalence of vitamin D deficiency/insufficiency and thus they may not have been able to detect small effects [29]. Evidence supports some risks at levels of circulating 25-OH-D above 32 ng/mL. For example, in white women, the risk of a small-for-gestational-age baby was the lowest between 24 and 32 ng/mL with a steep rise in risk at lower levels of 25-OH-D but also a more gradual increase in risk at higher values (there was no association among black women; [4]). Other epidemiological studies have found an association of increased risk at higher levels of circulating 25-OH-D for a number of pathologies both in and outside of pregnancy, including all-cause mortality [30], though, again, causality has not been shown [21, 30]. In most cases, the increase in risk at high levels is much less than the increase in risk for levels below 30 ng/mL. Finally, the fact that vitamin D levels in our ancient ancestors likely exceeded the IOM recommendations does not prove that those higher levels are conducive to better health and wellbeing in the modern environment.

The conflict between researchers and experts in vitamin D metabolism during pregnancy appears to be reflected in the clinical practice of obstetrician-gynecologists. About half of the respondents in this study appear to follow the recommendations from IOM and half are closer to the recommendations from the Endocrine Society. Because relatively few obstetrician-gynecologists in this study report that they routinely screen their pregnant patients, the difference in practice is primarily in the level of recommended vitamin D supplementation during pregnancy. Few reported recommending supplementation levels as high as those suggested by the Endocrine Society, but half would recommend levels above those of the IOM report.

The Endocrine Society considers pregnant and lactating women as high-risk groups for vitamin D insufficiency and recommends universal screening [19]. Screening does have a cost, however, and this study indicates that few obstetrician-gynecologists appear to consider universal screening appropriate. The majority of respondents would consider screening for specific patient conditions (e.g., gastric bypass surgery); a lesser proportion would consider screening because of obesity or for African American women despite both of these patient characteristics being known risk factors for inadequate vitamin D status. Dark skin pigmentation, as opposed to African heritage per se, is a risk factor for poor vitamin D status, especially in higher latitudes where sun intensity decreases outside of the summer months.

Caution should be exercised in extending the results of this study to US obstetrician-gynecologists in general, due to the small sample size. We are confident that the fact that the study only included CARN members was unlikely to affect the results, as this group is actively managed to preserve its demographic similarity to practicing ACOG Fellows as a whole. Over the last twenty years, we have found few instances where CARN Fellows' opinions differed from those of non-CARN Fellows when both groups were sent identical surveys. However, it remains a possibility.

## 5. Conclusions

US obstetricians appear cognizant of the importance of maternal vitamin D status during pregnancy. There is concern regarding vitamin D insufficiency within their pregnant patients. Although few screen most of their pregnant patients for vitamin D status, most would recommend vitamin D supplementation even without screening. Clinical practices regarding levels of supplementation and levels of maternal circulating 25-OH-D that would indicate increased concern are split, just as is opinion among experts and researchers. Better quality evidence is needed to inform practice. Ongoing clinical trials may provide the needed guidance, but until more definitive results are achieved clinical practice in the US will likely remain variable across physicians, with uncertain public health consequences.

## Figures and Tables

**Table 1:** Respondent demographic and practice data.

	Male (*n* = 43)	Female (*n* = 58)	*P* value
*Years in practice*	26.2 + 1.4 years	17.1 + 1.1 years	0.001

	Male (*n* = 41) 2 did not answer	Female (*n* = 57) 1 did not answer	

*Racial/ethnic background*			NS
Non-Hispanic white	34.7%	50.0%	
Non-Hispanic black	2.0%	1.0%	
Hispanic	1.0%	2.0%	
Asian/Pacific Islander	1.0%	2.0%	
Multiracial/other	2.0%	3.1%	

	Male (*n* = 41) 2 did not answer	Female (*n* = 58)	

*Practice setting*			0.073
Ob/Gyn partnership/group	19.2%	25.3%	
University faculty	3.0%	15.2%	
Multispecialty group	10.1%	6.1%	
Solo practice	7.1%	8.1%	
Other	2.0%	4.0%	

	Male (*n* = 41) 2 did not answer	Female (*n* = 58)	

*Practice location*			0.066
Urban inner city	7.1%	12.1%	
Urban noninner city	11.1%	24.2%	
Suburban	13.1%	16.2%	
Rural/midsized town	10.1%	6.1%	

**Table 2:** Potential sources of vitamin D that the responding physicians would and would not recommend to their patients. Totals do not add to 100% because respondents could answer that they were neutral.

Vitamin D source	Would recommend	Would not recommend
Fortified dairy products	84.2%	5.0%
Green leafy vegetables	77.2%	5.0%
Fatty fish	76.2%	4.0%
Fortified cereals	74.3%	7.9%
Vitamin D supplements	73.3%	12.9%
Multivitamins	71.3%	10.9%
Sun exposure	67.3%	18.8%
Fish oils	55.4%	10.9%
Mushrooms	18.8%	30.7%
Animal liver	11.9%	42.6%
Artificial UV light source	5.0%	62.4%

**Table 3:** The effect of the respondents' relative concern over vitamin D insufficiency among their patients on their opinions on vitamin D-related statements.

	Vitamin D insufficiency is not a problem in my patient population (*n* = 29)	Vitamin D insufficiency is a problem in my patient population (*n* = 66)
Vitamin D supplementation during pregnancy is safe	69.0% agree or strongly agree	93.9% agree or strongly agree

All pregnant women should be screened for vitamin D status	6.9% agree or strongly agree	30.3% agree or strongly agree

Vitamin D supplementation during pregnancy usually is not necessary	31.0% disagree or strongly disagree	66.7% disagree or strongly disagree

I am generally not concerned about vitamin D deficiency in my pregnant patients	20.7% disagree or strongly disagree	65.2% disagree or strongly disagree

Pregnant women taking prenatal vitamins are at low risk for vitamin D deficiency	48.3% disagree or strongly disagree	75.4% disagree or strongly disagree

Most of my pregnant patients will get enough vitamin D through sun exposure and diet	51.7% disagree or strongly disagree	75.8% disagree or strongly disagree

**Table 4:** Institute of Medicine and the Endocrine Society recommendations for vitamin D during pregnancy.

	Recommended daily allowance (RDA)	Daily requirement	Tolerable daily upper intake level	Minimal serum 25-hydroxyvitamin D level
*Institute of Medicine*				
14–18 yr	600 IU		4,000 IU	20 ng/mL (50 nmol/L)
30–50 yr	600 IU		4,000 IU	20 ng/mL (50 nmol/L)

*Endocrine Society*				
14–18 yr		600–1,000 IU	4,000 IU	30 ng/mL (75 nmol/L)
30–50 yr		1,500–2,000 IU	10,000 IU	30 ng/mL (75 nmol/L)
